# Gpr97 is essential for the follicular *versus* marginal zone B-lymphocyte fate decision

**DOI:** 10.1038/cddis.2013.346

**Published:** 2013-10-10

**Authors:** J-j Wang, L-l Zhang, Hong-x Zhang, C-l Shen, S-y Lu, Y Kuang, Y-h Wan, W-g Wang, H-m Yan, S-y Dang, J Fei, X-l Jin, Z-g Wang

**Affiliations:** 1 State Key Laboratory of Medical Genomics, Research Center for Experimental Medicine, Rui-Jin Hospital affiliated to Shanghai Jiao Tong University School of Medicine (SJTUSM), Shanghai, China; 2 Shanghai Research Center for Model Organisms, Shanghai, China; 3 Model Organism Division, Department of Medical Genetics, E-Institutes of Shanghai Universities, SJTUSM, Shanghai, China; 4 Department of Pathology, Shanghai Rui-Jin Hospital affiliated to SJTUSM, Shanghai, China

**Keywords:** *Gpr97*, knockout mice, B lymphopoiesis, follicular B cells, lambda 5 gene

## Abstract

Gpr97 is an orphan adhesion GPCR and is highly conserved among species. Up to now, its physiological function remains largely unknown. Here, we show that Gpr97 deficiency results in an extensive reduction in B220^+^ lymphocytes in mice. More intensive analyses reveal an expanded marginal zone but a decreased follicular B-cell population in *Gpr97*^*−/−*^spleen, which displays disorganized architecture characterized by diffuse, irregular B-cell areas and the absence of discrete perifollicular marginal and mantle zones. *In vivo* functional studies reveal that the mutant mice could generate antibody responses to T cell-dependent and independent antigens, albeit enhanced response to the former and weakened response to the latter. By screening for the molecular events involved in the observed phenotypes, we found that lambda 5 expression is downregulated and its upstream inhibitor *Aiolos* is increased in the spleen of mutant mice, accompanied by significantly enhanced phosphorylation and nuclear translocation of cAMP response element-binding protein. Interestingly, increased constitutive Nf-*κ*b p50/p65 expression and activity were observed in *Gpr97*^*−/−*^ spleen, implicating a crucial role of Gpr97 in regulating Nf-*κ*b activity. These findings uncover a novel biological function of Gpr97 in regulating B-cell development, implying Gpr97 as a potential therapeutic target for treatment of immunological disorders.

B cells develop from hematopoietic stem cells in the BM. Early development and commitment to the B-cell lineage depend on diverse transcription factors, including early B-cell factor, PU.1, E2A and PAX5.^1^ The earliest committed B-cell progenitors in the active cell cycle begin DJ rearrangement, followed by V(D)J rearrangement at the H-chain loci at the pro-B stage. After a productive V(D)J recombination, the cytoplasmic *μ* heavy chains (c*μ*HCs) are expressed and then paired with surrogate L chains (VpreB and *λ*5 proteins) to form the pre-B-cell receptor (BCR) at the pre-B-cell stage. Expression of the pre-BCR on the cell surface allows the clonal expansion and rearrangement of immunoglobulin-light chain (*IgL*) gene segments.^2^ The presence of surface IgM permits negative selection to occur (in which tolerance mechanisms delete, anergize or edit autoreactive clones).^3^ Naive B220^+^IgM^+^ B cells that survive negative selection then exit the marrow.

B cells continue their development in the spleen, where they pass through transitional stages transitional type 1 (T1) and transitional type 2 (T2) and are subjected to a further round of negative selection before becoming totally mature.^4^ The most prominent mature B cells in the spleen are follicular (FO) B cells, which continue circulating to the follicles in the spleen, to the lymph nodes and to the BM until they either die or encounter cognate antigen and undergo further maturation. Marginal zone (MZ) B cells, located at the outer rim of follicles, represent a smaller fraction of splenic B cells.^5^ The MZ and FO B-cell subsets differ significantly in phenotype, function and anatomical location. How these subpopulations are selected remains incompletely understood. Growing evidence has supported an important role for transcription factors in regulating MZ and FO B-cell fate. This is largely based on the observations that genetically mutant mice deficient in a series of transcription factors exhibited imbalanced development of MZ and FO B cells.^6, 7, 8, 9, 10, 11^

The adhesion GPCRs, which have 33 members in humans, contain large extracellular N-terminal domain containing a range of protein domains found in cell adhesion proteins, and C-terminal domain homologous to secretin-like GPCR.^12, 13, 14^ N- and C-terminal domains can be autocatalytically cleaved at the membrane-proximal GPCR proteolytic site domain, which is a characteristic feature of this receptor family.^15^ The majority of adhesion GPCRs are still orphans, for which neither ligand nor function is known. There is increasing evidence for the roles of adhesion GPCRs in the central nervous system, immune system and tumorigenesis. For example, CELSR1-3^16, 17^ and latrophilin1^18^ coordinate neuronal development and neurotransmitter release, respectively. CD97^19, 20^ and EMR1-3^21, 22, 23^ are involved in coordinating both the innate and the acquired immune responses. GPR124 promotes tumor angiogenesis.^24^ Although there is no consensus yet about the physiological functions of adhesion GPCRs and their molecular mechanisms of signaling, the existing data suggest that this receptor class mediates essential cell–cell and cell–matrix interactions.^12, 25^

GPR97 is an orphan adhesion GPCR with homology to the better characterized HE6 (human epididymis-specific protein 6) and GPR56 (human brain-specific protein). Like HE6 and GPR56, GPR97 possesses both an exceptionally long extracellular region, characteristic of cell adhesion proteins and an intracellular region reminiscent of other GPCRs.^26, 27^ GPR97 was found by searching human genome databases.^26^ Previous work revealed that *Gpr97* mRNA is highly expressed in immune cells.^28^ Other study has shown that GPR97 is coupled to G_o_, which means that the inactivation of GPR97 would lead to an increase in cAMP levels in target cells.^29^ To investigate the biological function of *Gpr97 in vivo,* we have generated *Gpr97*^*−/−*^ mice. The phenotypic analyses have demonstrated an indispensable role of Gpr97 in maintaining B-lymphocyte population, especially in regulating constitutive CREB and Nf-*κ*b activities.

## Results

### Expression profile of *Gpr97* in mice

To determine the expression pattern of *Gpr97* in normal adult mice, we performed semi-quantitative and real-time reverse transcription (RT)–PCR analyses on various mouse tissues. As shown in Supplementary Figure S1, the highest expression level of *Gpr97* mRNA was found in BM, and relatively low but detectable expression levels were also observed in heart, kidney and spleen tissues, implicating the tissue compartments where *Gpr97* could execute its physiological functions in mice.

### *Gpr97*^−/−^ mice are born alive and appear grossly normal

**Figure 1:**
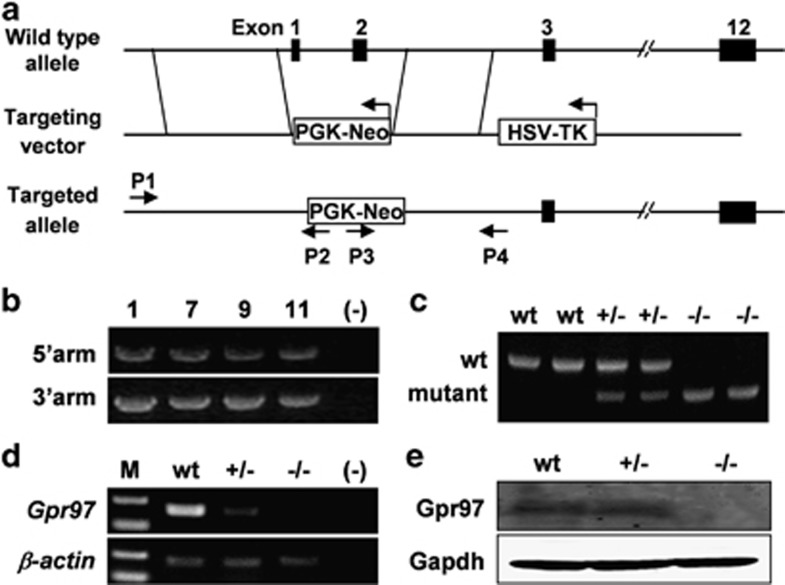
Targeted disruption of *Gpr97* in mice. (**a**) Schematic representation of *Gpr97* gene KO strategy. The targeting vector was designed to delete exon 1 harboring ATG codon and exon 2. Genomic regions amplified by PCR for genotyping are indicated by arrows. Exons are indicated as black boxes. (**b**) PCR analysis of ES cell clones. Genomic DNA extracted from ES clones was amplified using 5′-external and 3′-external primers as shown in panel (**a**). Homologous recombination events yielded a 6.5-kb or 3.8-kb fragment, respectively. (**c**) PCR genotyping of progenies from heterozygous matings. WT (+/+), heterozygous (+/−) and homozygous (−/−) mice were identified by PCR amplification of the fragments specific for either *Gpr97* WT allele (944-bp) or the mutant allele (730-bp). (**d**) RT-PCR analysis for *Gpr97* mRNA in BM of WT, *Gpr97*^+/−^ and *Gpr97*^−/−^ mice. The *Gpr97*-specific 192-bp product is absent in *Gpr97*^−/−^ and reduced in *Gpr97*^+/−^ mice. *β*-actin was used as a loading control. (**e**) Immunoblotting of BM protein samples with antibodies against Gpr97 and Gapdh shows absence of Gpr97 in mutant mice

To explore the physiological function of *Gpr97 in vivo*, we generated a mouse model with global-targeted deletion of *Gpr97* (Figure 1a). The homologous recombination in ES cells was confirmed (Figure 1b), and the genotypes of mice were verified by PCR analysis of genomic DNA (Figure 1c). The absence of *Gpr97* expression was confirmed by analyzing the *Gpr97* mRNA by RT-PCR (Figure 1d) and protein expression by western blot analysis (Figure 1e). As expected, *Gpr97* mRNA and protein were disrupted in the BM of *Gpr97*^*−/−*^ mice. However, the mutant mice were viable and reached the adult stage without any gross developmental abnormalities, suggesting that *Gpr97* is not indispensible for normal development.

### Reduction of B-cell population in *Gpr97*^*−/−*^ mice

**Figure 2:**
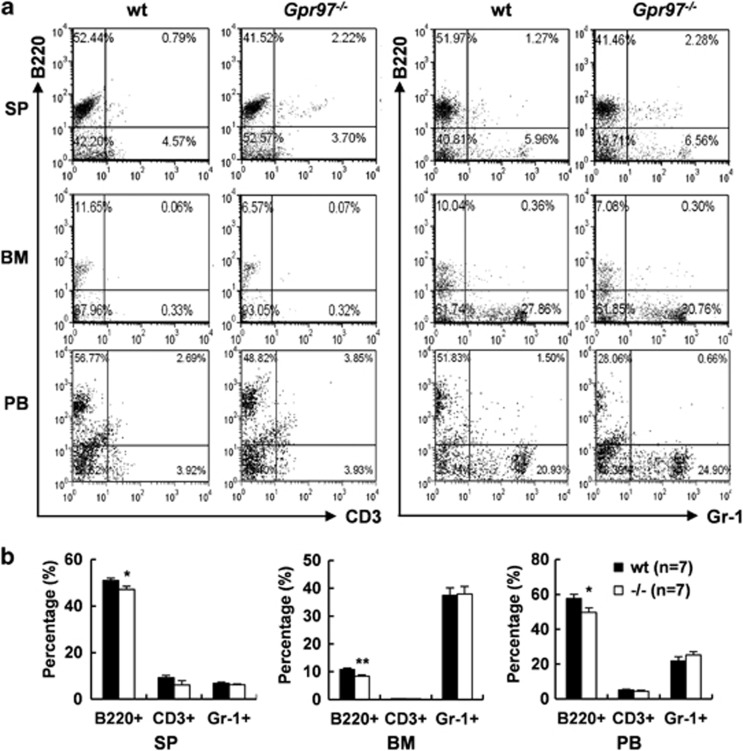
Absence of *Gpr97* impairs B lymphopoiesis in BM, spleen (SP) and peripheral blood (PB). (**a**) Splenocytes, BM and PB cells were recovered from 12-week-old WT and age-, sex-matched KO mice (*n*=7 for each). The cells were labeled with monoclonal antibodies as indicated and subjected to flow cytometric analysis. The numbers in each quadrant indicate the percentages of cell populations. (**b**) The relative numbers of B220^+^, CD3^+^ and Gr-1^+^ cells were expressed as mean±S.E.M. (*n*=7). Individual fractions defined in (**a**) were calculated. *P<0.05; **P<0.01

Considering the features of *Gpr97* expression profile in mice, we performed several phenotypic screening tests for detection of some parameters potentially affected due to *Gpr97* deficiency. It was found that no significant differences in body weight, ratios of thymus or spleen weight to body weight, as well as bone mineral density between sex- and age-matched WT and *Gpr97*^*−/−*^ mice (Supplementary Figure S2a-d). Hematological examination revealed that the numbers of white blood cells, red blood cells and platelets remained unaffected in PB of *Gpr97*^*−/−*^ mice (Supplementary Figure S2e). The differential counting of BM cells was performed by flow cytometry using lineage-specific cell-surface markers. The results showed that the percentages of CD3^+^, Ter119^+^, CD41^+^ and Gr-1^+^ cells were comparable between WT and *Gpr97*^*−/−*^ mice but B220^+^ cell population was decreased in *Gpr97*-deficient BM cells (Supplementary Figure S2f). More intensive analysis revealed that the reduction of B220^+^ cells was observed not only in BM but also in spleen and PB of the mice lacking *Gpr97* as compared with WT littermates (Figure 2a). The percentages of T cells and granulocytes were not significantly different between WT and *Gpr97*^*−/−*^ mice as judged by CD3 or Gr-1 staining, respectively (Figure 2b). These results suggested that Gpr97 is essential in maintaining B220^+^-cell population. To address whether the reduction of B lymphocytes was due to impaired cell proliferation or increased apoptosis, single-cell suspensions of splenocytes were cultured with or without LPS (20 *μ*g/ml) stimulation. MTT assay revealed that *Gpr97*^*−/−*^splenocytes proliferated as rapidly as WT cells upon LPS treatment (data not shown). However, annexin V/PI staining and FACS analyses showed that B220^+^ cells deficient for *Gpr97* had increased apoptosis when compared with WT cells cultured for 24 h in the presence or absence of LPS (Supplementary Figure S3). We also analyzed *Bcl-2*, *Bax* and caspase-3 mRNA levels, as well as caspase-3 activity in splenic B220^+^ cells, and found that *Bcl-2* expression level is decreased and caspase-3 activity is increased in *Gpr97*^*−/−*^splenic B lymphocytes cultured for 24 h with or without LPS (Supplementary Figure S4). Taken together, these observations demonstrate that the increased apoptosis of B220^+^ cells in *Gpr97*^*−/−*^spleen was attributed to enhanced caspase-3 activity.

### Abnormal B-cell subpopulations in BM and spleen of *Gpr97*^*−/−*^ mice

**Figure 3:**
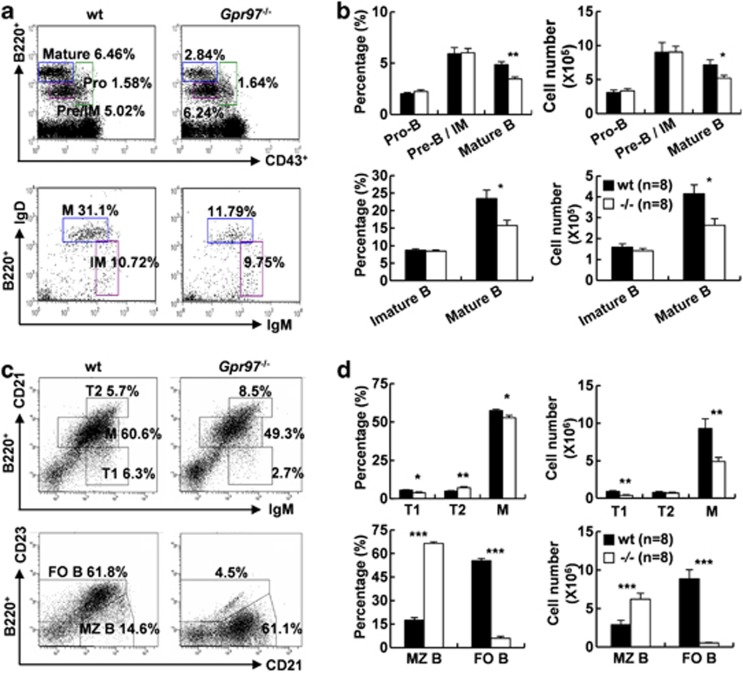
Analysis of subpopulations of B cells from WT and KO mice. (**a**) BM cells from WT and *Gpr97*-KO mice (*n*=8 for each) were labeled for B220 and CD43 (upper panels), or B220, IgM, IgD (lower panels), and gated on total lymphocytes or B220^+^ cells, respectively. Numbers represent the percentages of cells in the plot that fall within the different regions, corresponding to different stages of B-cell differentiation. (**b**) The relative and absolute numbers of CD43^+^B220^int^ (pro-B), CD43^−^B220^int^ (pre-B/immature B), CD43^−^B220^hi^ (mature B), B220^+^IgM^+^IgD^−^ (immature B), B220^+^IgM^+^IgD^+^ (mature B) in BM were expressed as mean±S.E.M. (*n*=8). Individual fractions defined in (**a**) were calculated. (**c**) Flow cytometry profiles of the spleen of WT and KO mice labeled for B220, IgM, CD21 (upper panels), or B220, CD21, CD23 (lower panels), and gated on B220^+^ cells. Numbers represent the percentages of cells in the plot that fall within the different regions, corresponding to different stages of B cell differentiation. (**d**) The bar charts summarize the relative and absolute numbers of B220^+^CD21^low/−^IgM^high^ (T1), B220^+^CD21^high^IgM^high^ (T2), B220^+^CD21^int^IgM^int^ (mature B, M), B220^+^CD21^high^CD23^low/−^ (MZ B), B220^+^CD21^int^CD23^high^ (FO B) in the spleen. Individual fractions defined in (**c**) were used for calculations. Data show the mean±S.E.M. The differences between WT and KO mice were examined for statistical significance that was indicated as asterisks: *P<0.05; **P<0.01; ***P<0.001

To further characterize the reduction of B-cell population in *Gpr97*-deficient mice, we performed an extensive survey of surface marker expression on B-lineage cells in BM.^30, 31^ It was found that mature B (B220^+^IgM^+^IgD^+^, M)-cell populations were reduced in *Gpr97*^*−/−*^ mice as compared with WT controls. The percentages and absolute cell numbers of mature B cells were reduced by 32.7% and 36.2%, respectively. Although pro-B (CD43^+^B220^int^), pre-B/immature B (CD43^−^B220^int^) and immature B (B220^+^IgM^+^IgD^−^, IM) cell populations in *Gpr97* mutant BM were comparable to those found in WT littermates (Figures 3a and b). These findings suggest that *Gpr97* is not involved in early B-lineage cell commitment but is required for development of mature B cells in secondary lymphoid tissues.

By analyzing the splenic B-cell compartment, we observed that the percentages of mature B (B220^+^CD21^int^IgM^int^) and T1 B (B220^+^CD21^low/−^IgM^high^)^32^ cells in *Gpr97* mutant spleens were lower than those in WT controls (Figure 3c, top; 3d, top left). Accordingly, the absolute numbers of mature B and T1 B cells were significantly reduced due to a decrease in total splenic B cells in mutant mice as compared with WT controls (Figure 3d, top right). Meanwhile, the T2 B (B220^+^CD21^high^IgM^high^)^33^ cells of mutant mice were found significantly increased with regard to percentages, but no significant changes were observed in the absolute cell numbers (Figure 3c, top; 3d, top right). By means of flow cytometry, mature B cells in spleen were separated into MZ B and FO B cells in terms of the surface expression of CD21 and CD23^34, 35^ (Figure 3c, bottom). Our results revealed that MZ B cells (B220^+^CD21^high^CD23^low/−^) were markedly increased by 3.8-fold in percentage and 2.5-fold in absolute numbers after *Gpr97* deletion, whereas FO B cells (B220^+^CD21^int^CD23^high^) were markedly decreased by 9-fold in percentage and 10-fold in absolute numbers (Figure 3d, bottom).

### *Gpr97*^*−/−*^ mice display abnormal splenic architecture

**Figure 4:**
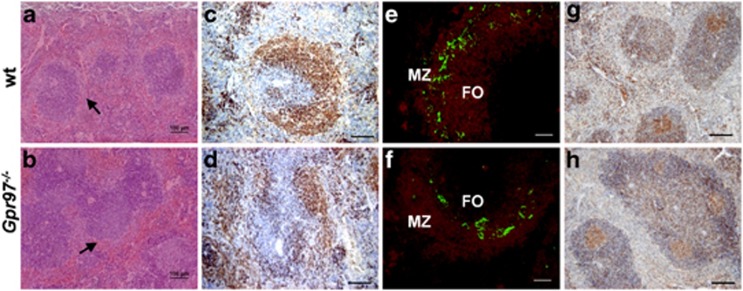
Histopathological and immunohistochemical analysis of the spleen of WT and KO mice (*n*=4, each group). (**a**) and (**b**) Spleen sections from 12-week-old WT and KO mice were stained with H&E. Perifollicular MZs were indicated by arrows. Scale bar, 100 *μ*m. (**c**) and (**d**) Immunohistochemistry of spleen sections from (**a**) and (**b**) were stained with B cell-specific mAb B220. Scale bar, 200 *μ*m. (**e**) and (**f**) Histological section of *Gpr97*-KO spleen showed an increased population of IgM^+^ cells (labeled in red with anti-IgM Ab) in MZ. The green signals represent FITC-labeled anti-MOMA-1, which is specific for metallophilic macrophages that separate the MZ from the follicles. Scale bar, 200 *μ*m. (**g**) and (**h**) Mice were killed 1 week after secondary immunization with IFA-OVA. Sections of spleen were labeled with PNA (brown). Hematoxylin counterstain was used. Scale bar, 200 *μ*m

Microscopic evaluation of the spleen of *Gpr97*^*−/−*^ mice showed a disruption of normal architecture characterized by diffuse, irregular follicular (B-cell) areas with an absence of a discrete perifollicular MZ (Figures 4a and b). These morphological abnormalities were substantiated after immunohistochemical detection of B cells (Figures 4c and d). To corroborate the proportional differences in the mature B-cell populations between *Gpr97*^*−/−*^ and WT controls, histochemical analysis was performed. Sections were stained with anti-IgM for B cells, and MOMA-1 for metallophilic macrophages to delineate the border between the MZ and the follicular area (Figures 4e and f). Consistent with the increased numbers of MZ B cells observed in flow cytometry, the MZ area of *Gpr97*^*−/−*^ mouse spleen was larger than that of WT controls.

The abnormal architecture of *Gpr97*^*−/−*^ spleen suggested that immune responses depending on cellular interactions in the follicle might not be fully functional. To test this hypothesis, the germinal centers (GCs) were examined in KO and WT mice after secondary immunization. GCs are typical structures formed in secondary lymphoid organs during the course of a TD immune response, and are the place where affinity maturation, class switching and memory cell formation take place.^36^ Spleen sections were stained with peanut agglutinin (PNA) for the presence of GCs (Figures 4g and h). The results revealed that PNA-binding GC B cells clearly developed in the spleen of *Gpr97*-KO mice, which indicated an intact GC formation despite *Gpr97* deletion.

### Impaired immunoglobulin production, humoral primary and secondary immune responses in *Gpr97*^*−/−*^ mice

**Figure 5:**
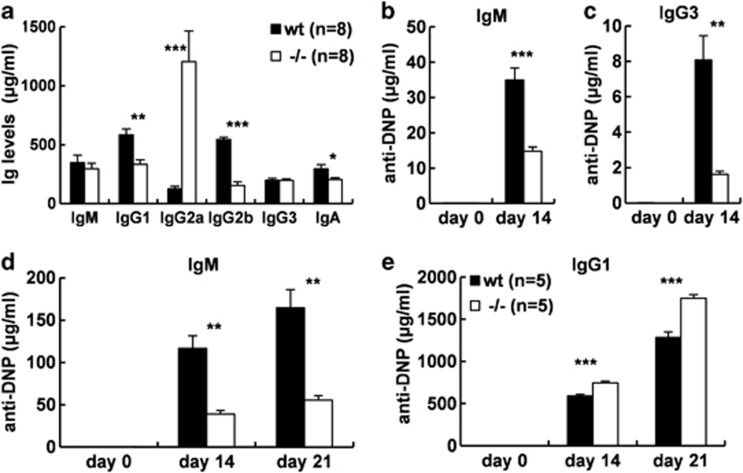
Serum Ig levels, TI and TD antigen responses in WT and KO mice. (**a**) IgM, IgG1, IgG2a, IgG2b, IgG3 and IgA levels in the sera of 3-month-old WT (*n*=8) and KO (*n*=8) mice were evaluated by ELISA. Serum levels of IgM (**b**) and IgG3 (**c**) anti-DNP antibodies were measured before (*t*_0_) and 14 days after (*t*_14_) immunization with the TI antigen DNP-Ficoll in WT and KO mice. Serum levels of IgM (**d**) and IgG1 (**e**) anti-DNP antibodies were measured before (*t*_0_), at the time of secondary immunization (*t*_14_) and 7 days after secondary immunization (*t*_21_) with the TD antigen DNP-KLH in WT and KO mice. Data show the mean±S.E.M. The differences between WT and KO mice were examined for statistical significance that was indicated as asterisks: *P<0.05; **P<0.01; ***P<0.001

To determine whether *Gpr97* deficiency had an effect on Ig production, Ig titers were assessed in 3-month-old WT and *Gpr97*^*−/−*^ mice by ELISA. As shown in Figure 5a, levels of IgG1, IgG2b and IgA were significantly decreased, whereas IgG2a level was significantly increased in KO mice as compared with WT controls. IgM and IgG3 levels were similar between WT and KO mice. Next, we analyzed the ability of *Gpr97*-KO mice to mount antibody responses to TI and TD antigens. At day 14 after immunization with TI antigen DNP-Ficoll, production of both IgM and IgG3 anti-DNP antibodies was reduced in *Gpr97*-KO mice as compared with WT controls (Figures 5b and c). The amount of DNP-specific IgM antibodies after TD immunization was lower in *Gpr97*-KO mice than that in controls. Level of DNP-specific IgG1 instead was higher in mutant mice after TD immunization (Figures 5d and e). These data indicate that *Gpr97*-deficient animals are able to make both TD primary and secondary immune responses, consistent with the data of the GC formation in *Gpr97*-KO mice, as well as TI responses, although at reduced levels compared with WT animals.

### Downregulation of lambda 5 expression in *Gpr97*-deficient mice

**Figure 6:**
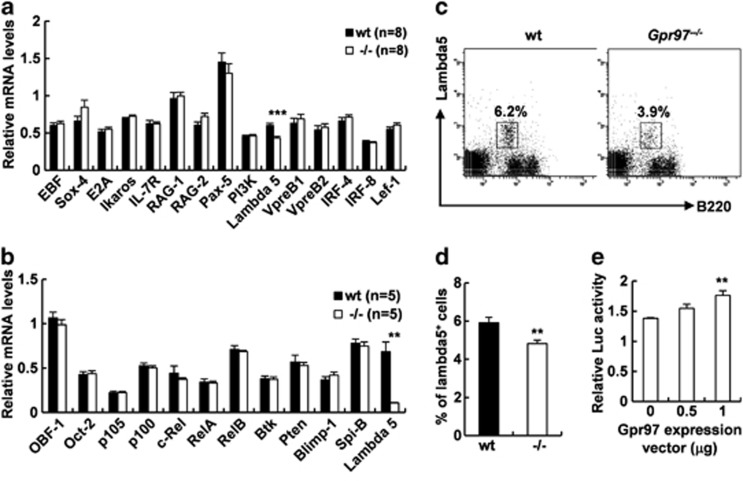
*Gpr97* deficiency leads to downregulation of *lambda 5*. (**a**) The expression levels of the genes related to B-cell development were evaluated by real-time qPCR in BM cells. There was a reduction in *lambda 5* mRNA level in KO mice as compared with WT mice (*n*=8 for each group). (**b**) Real-time qPCR analysis of the expression levels of genes in splenocytes. *Lambda 5* mRNA level was reduced in KO mice as compared with WT mice (*n*=8 for each group). (**c**) Staining of BM cells from WT and KO mice (*n*=7 for each group) with anti-B220 and anti-lambda 5 antibodies. (**d**) Relative numbers of lambda 5^+^ cell were presented as mean±S.E.M. (*n*=7). (**e**) *Lambda 5* reporter construct and *Gpr97* expression vector were co-transfected into Hela cells. Relative luciferase activity was monitored in triplicate 48 h post transfection. The differences between two groups were examined for statistical significance that was indicated as asterisks in panels (**a**), (**d**) and (**e**): **P<0.01; ***P<0.001

The observation that abnormal B-cell development in KO mice prompted us to evaluate the expression pattern of a multitude of different transcriptional factors by real-time PCR, which have been linked to distinct stages of the life of B lymphocytes, such as differentiation in the BM, migration to the peripheral organs and antigen-induced activation.^37, 38^ The mRNA expression levels of several genes were found unaltered as compared with those in WT control except for lambda 5 in both BM (Figure 6a) and spleen (Figure 6b). The lambda 5 transcript is reduced by 27.8% and 84.1% in BM and spleen of *Gpr97*^*−/−*^ mice, respectively. To confirm this observation, we carried out flow cytometric analysis to examine the percentage of lambda 5-positive cells in BM. The results showed that the percentage of lambda 5-positive cells was reduced by 18.6% in BM of *Gpr97*^*−/−*^ mice as compared with WT controls, and the difference was statistically significant (Figures 6c and d). To further investigate the relationship between *Gpr97* and *lambda 5*, reporter construct with *lambda 5* promoter inserted upstream of the luciferase coding region in the pGL3 luciferase reporter vector and *Gpr97* expression vector were transiently co-transfected into Hela cells, and results showed that luciferase activity in Hela cells increased with dose of Gpr97 expression vector, as compared with those co-transfected with pcDNA^TM^3.1/*myc*-His (−) B control vector (Figure 6e). These data suggest that *Gpr97* could positively regulate *lambda 5* expression at transcriptional level.

### A crucial role of Gpr97 in regulating CREB signaling pathway

**Figure 7:**
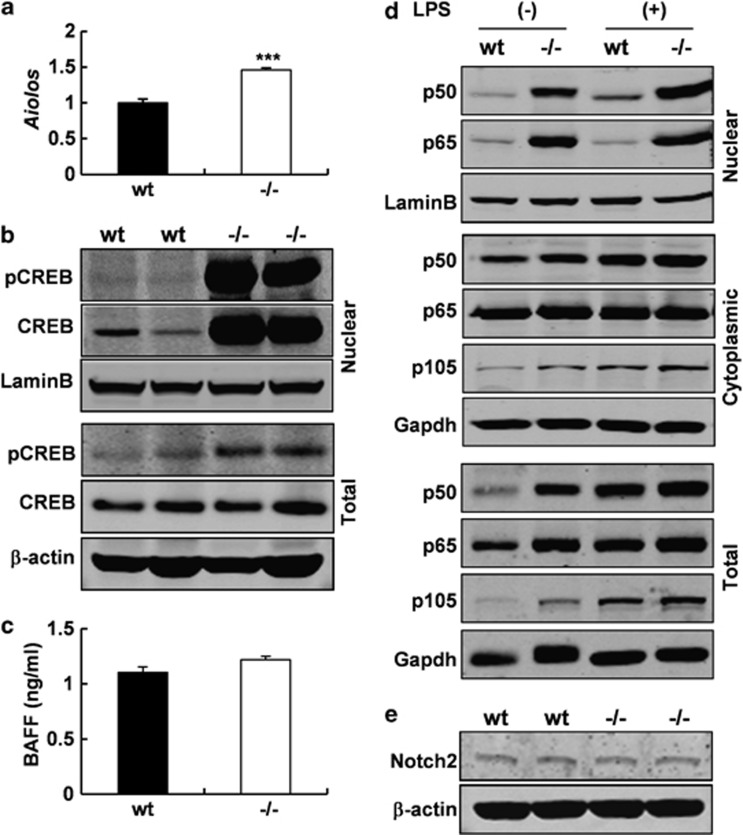
pCREB and *Aiolos* levels were increased in the spleen of *Gpr97*-KO mice. (**a**) Real-time qPCR analysis of *Aiolos* expression levels in splenocytes (*n*=5 for each group). Data show the mean±S.D. (**b**) Western blot images representing the expressions of pCREB and total CREB in total cell lysates and nuclear fractions from spleen. (**c**) No difference was observed in serum BAFF level between WT and KO mice (*n*=5, each group). Data show the mean±S.E.M. (**d**) Immunoblots of NF-*κ*B1/p105/p50 and p65 in whole-cell lysates, cytoplasmic and nuclear fractions from splenocytes. (**e**) Notch2 protein expression was analyzed in spleen by western blot. The differences between WT and KO mice were examined for statistical significance that was indicated as asterisks: ***P<0.001

Given that Gpr97 is an orphan GPCR, we presumed that Gpr97 would be more likely to regulate lambda 5 indirectly. Through a literature search, we found that Aiolos, a member of the Ikaros family of transcription factors, is required for the efficient silencing of lambda 5.^39^ To determine whether the decreased level of lambda 5 is due to an increased Aiolos level in *Gpr97*^*−/−*^ mice, we analyzed the Aiolos level in the spleen by real-time PCR. As expected, an increased *Aiolos* expression level was observed in the spleen of KO mice as compared with WT controls (Figure 7a). Then, we analyzed the promoter of *Aiolos* by TFSEARCH software (Computational Biology Research Center, Tsukuba, Japan) for the purpose of finding potential transcription factor binding sites. As predicted, a CREB-binding site was identified in *Aiolos* promoter region. The transcription factor CREB is one of the components of many signaling cascades, and is regulated by the GPCR–cAMP–PKA pathway. Thus, we examined the levels of pCREB and total CREB in the spleen of *Gpr97*^*−/−*^ mice by western blot analysis. As shown in Figure 7b, the level of pCREB in total cell lysate from the spleen of KO mice was found increased while CREB level remains comparable to that of WT control. Most strikingly, both CREB and pCREB levels in the nuclear extract were found dramatically increased as compared with WT controls, suggesting that Gpr97 could negatively regulate CREB signaling. Thus, we believe that the ablation of *Gpr97* leads to increased phosphorylation of CREB, and the increased level of pCREB may enhance the expression level of *Aiolos*, which downregulates the expression of lambda 5.

### Gpr97 negatively regulates Nf-*κ*b signaling

BM-derived B cells make an important cell fate choice to develop into either FO B cells or MZ B cells in the spleen, which depends on signaling through BCR, Notch2, the receptor for B cell-activating factor and the canonical nuclear factor-*κ*B (NF-*κ*B) pathway, as well as signals involved in the migration and anatomical retention of MZ B cells.^40^ Hence, it is possible that *Gpr97* deletion might affect BAFF, NF-*κ*B or Notch2 expression in spleen. This possibility was elucidated by the following three experiments. First, we examined the serum BAFF level by ELISA, and found there is no difference between the two groups (Figure 7c). Second, western blot analysis showed that constitutive p105, p65 and p50 protein levels were increased to some extent in the total cell lysates of *Gpr97*^*−/−*^ splenocytes when compared with that of WT control. Accordingly, higher levels of p65 and p50 in the nuclear extracts of *Gpr97*^*−/−*^ splenocytes were observed with comparable levels of p65, p50, as well as p105 proteins in cytoplasmic fraction and responses to LPS stimulation between two groups (Figure 7d). These results indicate that Gpr97 deficiency leads to increased constitutive expression and activation of Nf-*κ*b components examined, suggesting that Gpr97 may negatively regulate Nf-*κ*b signaling. Third, the level of Notch2 in the spleen had no significant difference between WT and mutant mice, as demonstrated by western blot analysis (Figure 7e).

## Discussion

A previous study has established a Gpr97-deficient mouse model, which displays no any significant quantitative or qualitative defects in B- and T-cell development.^41^ Such discrepancy may attribute to the different mouse genetic background to that in this study. The mice used in the previous study have a homogenous 129Sv genetic background. However, the mutant mice used in this study are maintained in a mixed 129Sv/C57BL/6 background. Many studies have already addressed that genetic background is of great importance to immune research.^42, 43^ Through intensive phenotypic analysis, we have demonstrated that Gpr97 has a crucial role in maintaining normal splenic architecture, regulating B-lymphocyte development, specifically follicular B-cell development. To understand the molecular events underlying the impaired B-cell development, we have tested the expression levels of multiple transcription factors which have been demonstrated to have a role in B-cell development. For example, E2A and EBF control the immunoglobulin-gene rearrangement,^44^ Blimp1 is induced upon terminal differentiation to plasma cells^45^ and NF-*κ*B is described as a nuclear transactivator of the immunoglobulin-light chain enhancer and a cytokine-inducible transcription factor governing the expression of an important set of genes involved in inflammation and cell survival.^37^ Lambda 5, a component of pre-BCR, is expressed in B-cell development before conventional L chains. Deficiency of lambda 5 leads to a block in B-cell development in BM at the pre-B-cell stage, resulting in a marked decrease in the number of mature B cells in the periphery. The proportion was increased but not the absolute number of MZ B cells, whereas FO B cells were decreased. Lambda 5 KO mice are able to mount not only primary but also secondary TD and TI responses, albeit at reduced levels,^46, 47^ similar to the phenotypes observed in *Gpr97*^*−/−*^ mice.

To understand the relationship between Gpr97 and lambda 5, we discovered that the expression level of *Aiolos* which is required for silencing lambda 5, was significantly increased in the spleen of *Gpr97*^*−/−*^ mice. Interestingly, the promoter of *Aiolos* has a CREB-binding site predicted by TFSEARCH software. CREB, a cellular transcription factor, is activated by phosphorylation at Ser-133.^48^ The kinase responsible for this activating phosphorylation was identified as cAMP-dependent protein kinase, PKA. PKA activity is regulated by molecules that can alter cAMP levels, and hence by GPCRs, which regulate adenylate cyclase activity. Based on the previous findings, we examined the phosphorylation level of CREB in the *Gpr97*^*−/−*^ mice and found an increased phosphorylation level of CREB in both nuclear extracts and whole-cell lysates from the spleen of *Gpr97*^*−/−*^ mice. This observation is in accordance with the previous finding that *Gpr97* is coupled to G_o_, which means that the inactivation of *Gpr97* would lead to an increase in cAMP levels in target cells. According to these results, it is reasonable that Gpr97 modulates the expression of lambda 5 via Aiolos. We have noted that the follicular type I B-cell population was preserved in *Aiolos*-deficient mice, in which the strength of BCR signaling was increased, but the number of splenic MZ B cells was markedly decreased, as well as MZ precursor B cells. When the increased BCR signal strength in *Aiolos*-deficient mice was abrogated by crossing these mice with Xid mice, no less of MZ or MZ precursor B cells was observed, which indicates that increased BCR signaling hampers the differentiation of maturing B cells into MZ B cells and *Aiolos* was not directly required for MZ B-cell development.^49^ Taken together, these observations suggest that an increased *Aiolos* level may partially contributes to the enhancement of MZ B-cell development in *Gpr97*^*−/−*^mice.

There are several mechanisms that may explain the cell fate determination to develop into either FO B cells or MZ B cells in the spleen. First, BCR signaling strength is reported to have a critical role in mature B-cell fate decision. FO B-cell development is dependent on strong BCR signaling, whereas signals driving MZ B-cell development are weak.^40^ Second, the Notch signaling pathway is essential for mature B-cell fate determination. The mice lacking *Notch2*,^50^ *Rbpj*^51^ or *Dll1*^52^ have defects in MZ B-cell development. Third, BAFF and NF-*κ*B are also required for peripheral B cell development. In IL-7 transgenic mice which have a dramatic increase in the FO B-cell compartment,^53^ MZ B-cell number was reduced probably due to lower overall levels of BAFF. MZ B-cell development is defective in mice that lack p50.^54^ The absence of Rel and p65 also affects MZ B-cell development partially, which indicates that p50-Rel and p65-Rel complexes might be required for MZ B-cell development. Accordingly, we examined the levels of BAFF, NF-*κ*B and Notch2 in the spleens of WT and KO mice, and found that nuclear p50 and p65 were markedly increased in *Gpr97-*null mice, whereas serum BAFF and Notch2 proteins had no differences between WT and KO mice. These findings suggest that the enhanced Nf-*κ*b signaling strength may contribute to an increase in MZ B cells in *Gpr97*^*−/−*^ mice, whose phenotype is opposite to that of *p50*^*−/−*^ or *p65*^*−/−*^ mice. The question is how Gpr97 affects the levels of nuclear p50 and p65 in splenocytes. A possible inference is that PKA activation is able to simulate PKC and p38 MAPK, leading to IKK-dependent NF-*κ*B activation.^55^

In summary, our study uncovers a novel biological function of Gpr97 in regulating B-cell development, especially CREB and NF-*κ*B signaling pathways, implying Gpr97 as a potential therapeutic target for treatment of immunological disorders.

## Materials and Methods

### Mice

The mutant mice were generated by using a homologous recombination method and maintained on a mixed 129Sv/C57BL/6 background and housed under specific pathogen-free conditions at a constant room temperature of 22–24 °C with a 12-h light/dark cycle. Animal protocols and experiments were approved by the Animal Use and Care Committee of Shanghai Research Center for Model Organisms (Permit Number: 20110007). Age-matched littermates (12-week old) with different genotypes were used for phenotypic analyses. More detailed description on Materials and Methods can be found in the Supplementary Information on line.

### Immunization and determination of serum Ig levels

Titers of Ig in serum were tested using ELISA. In brief, 96-well plates were coated with goat anti-mouse Ig capture antibody overnight at 4 °C, followed by incubation with diluted serum samples and developed with HRP-conjugated goat anti-mouse isotype-specific antibodies and substrate ABTS (Southern Biotechnology, Birmingham, AL, USA). Reactions were read at 405 nm at 10 min after substrate addition using a microplate reader (BioTek, Winooski, VT, USA). To elicit TI antigen responses, gender-matched 12-week-old WT and KO mice were intraperitoneally injected with 50 *μ*g DNP-conjugated Ficoll (Biosearch Technologies, Petaluma, CA, USA). Sera were collected at the time of immunization and 14 days later, and anti-DNP IgM and IgG3 antibody titers were measured by ELISA. For TD antigen responses, 12-week-old WT and KO mice were intraperitoneally injected with 100 *μ*g DNP-keyhole limpet hemocyanin (KLH, Biosearch Technologies) with CFA. After 2 weeks, mice were injected again with 10 *μ*g DNP- KLH with IFA. Sera were collected on the day of the primary immunization, at the time of the secondary immunization, and another week later. Antibodies to DNP were measured by ELISA using plates coated with DNP-BSA (Biosearch Technologies).

### Cell culture

Suspensions of splenocytes (after isotonic erythrocyte lysis) were cultured in complete RPMI 1640 medium containing 10% FBS, 1% HEPES, 1% L-glutamate, 1% pen/strep and 0.1% 2-mercapthoethanol with or without LPS (20 *μ*g/ml) for 24 or 48 h. Monitoring of apoptosis or necrosis was performed by staining with annexin V and PI (Invitrogen, Grand Island, NY, USA) according to the manufacturer's instructions. Cells negative for PI but positive for annexin V were defined as apoptotic cells. Late apoptotic and necrotic cells were identified by strong PI staining.

### Histological analysis

Spleens were fixed for 24 h in 10% neutral buffered formalin, embedded in paraffin, sectioned (5 *μ*m) and then stained with hematoxylin and eosin. For immunohistochemistry, splenic sections were incubated with the antibodies against B220 (BD Pharmingen, Franklin Lakes, NJ, USA) in blocking buffer overnight at 4 °C. After washing, the sections were stained with biotin-labeled rat second antibody (KPL), and label developed using an ABC kit (Vector Laboratories, Burlingame, CA, USA). For immunofluorescence, spleens were frozen in Tissue-Tek OCT compound (Sakura, Torrance, CA, USA) and sectioned at a thickness of 8 *μ*m. Sections were then fixed for 15 min in 4% paraformaldehyde (PFA) and stored at −20 °C. For labeling, slides were washed, incubated for 10 min in PBS containing 0.25% Triton X-100 and blocked for 30 min in 1% BSA in PBS. Then sections were labeled overnight at 4 °C with primary antibody in blocking buffer. After extensive washing in PBS, slides were mounted in Antifade Mounting Medium (Beyotime, Shanghai, China). Primary antibodies used: FITC-MOMA-1 (AbD Serotec, Oxford, UK) and Texas Red-IgM (Vector Laboratories). GC formation in spleen was examined by histological analysis. Sections were labeled with PNA (Vector Laboratories), and color was developed using an ABC kit (Vector Laboratories).

### Analyses of mRNA expression

Total RNA was prepared from different mouse tissues using Trizol Reagent (Invitrogen) according to manufacturer's instructions. mRNA expression levels were assayed by semi- or real-time quantitative RT-PCR. The expression level of *β-actin* was used as an endogenous control. Real-time PCR analysis was performed using SYBR Premix Ex Taq kit (Takara, Dalian, China) on an Eppendorf Mastercycler system according to the manufacturer's protocol. All samples were tested in triplicate, and the results were normalized to *β-actin* expression.

### Flow cytometry

Single-cell suspensions of BM cells, splenocytes or PB cells were stained using standard procedures. Cells were adjusted to 2 × 10^7^ cells/ml in PBS with 2% FBS and 0.1% sodium azide, and 1 × 10^6^ cells were incubated 30 min on ice with appropriately diluted antibodies (1 ng/*μ*l) in a total volume of 100 *μ*l. After washing three times, the stained cells were analyzed using a flow cytometer (BD FACSAria, BD Pharmingen). Processed samples were analyzed using CellQuest software (BD Biosciences).

### Western blot analysis

Whole-cell lysates from BM cells (1–2 × 10^7^) were extracted with RIPA lysis buffer (Thermo Scientific, Waltham, MA, USA). Nuclear protein extracts from spleen were prepared according to the handbook of NE-PER Nuclear and Cytoplasmic Extraction Reagents (Thermo Scientific). The protein concentration was determined using the BCA Protein Assay Kit (Pierce, Rockford, IL, USA). Equal amounts of protein (60–80 *μ*g) were separated and transferred onto polyvinylidene fluoride membrane by semi-dry blotting. The membranes were incubated overnight with the antibodies listed in Supplementary Information. Infrared fluorescence on membranes was detected using an Odyssey infrared imaging system (LI-COR). For quantitative determinations, a densitometric analysis of signal bands was performed using Gel-Pro Analyzer software (Media Cybernetics. Bethesda, MD, USA).

### Statistical analysis

Quantitative results were presented graphically as mean±S.E. The statistical differences in the observed data were compared by one-way ANOVA (analysis of variance). *P*<0.05 was considered statistically significant.

## Abbreviations

GPCR: G protein-coupled receptor
FO: follicular
MZ: marginal zone
BM: bone marrow
BMD: bone mineral density
PB: peripheral blood
GC: germinal center
PNA: peanut agglutinin
CFA: complete Freund's adjuvant
IFA: incomplete Freund's adjuvant
