# Supplementary material for: Gpr97 is essential for the follicular versus marginal zone B-lymphocyte fate decision
Source: Cell Death Dis. 2013 Oct 10;4(10):e853–. doi: 10.1038/cddis.2013.346 (PMC3824656; doi:10.1038/cddis.2013.346)
Supplement: Supplementary Information [file cddis2013346x1.doc]

**Supplementary Information**

1. **Materials and Methods**

**Mice**

A targeting vector was constructed by replacing the mouse *Gpr97* genomic 3368-bp fragment covering exons 1 and 2 with the 1904-bp PGK-neo cassette for positive selection, and using a HSV-TK for negative selection. The targeting vector was introduced into embryonic stem (ES) cells derived from 129Sv. The ES cells harboring the homologous recombination were determined by PCR using two pairs of primers with direction and position depicted in Figure 1A. The sequences of primers used for genotyping are as follows: P1, 5′- GGCCTCTTCAGTCTGGTGTTC-3′; P2, 5′- GGCCTACCCGCTTCCATTGCTC-3′. P3, 5′-CCGTGCCTTCCTTGACCCTGG-3′; P4, 5′- CAGGAATGGACCGTAAGAGCAC-3′. The mutant mice were generated by using a homologous recombination method. One of 23 recombined clones was microinjected into blastocysts and implanted into pseudopregnant female recipients. Ten male chimeric mice were mated to WT C57BL/6 females. The F1 mice with germline transmission of the *Gpr97* KO allele were bred to F2 to produce homozygous KO mice. The mutant mice were maintained on a mixed 129Sv/C57BL/6 background and housed under specific pathogen-free (SPF) conditions at a constant room temperature of 22-24°C with a 12-h light/dark cycle. Animal protocols and experiments were approved by the Animal Use and Care Committee of Shanghai Research Center for Model Organisms (Permit Number: 20110007). Age-matched littermates (12-week-old) with different genotypes were used for phenotypic analyses.

**Mouse immunization**

CFA-OVA and IFA-OVA were prepared by coupling ovalbumin (OVA) (Sigma) with Complete Freund's Adjuvant (CFA) or Incomplete Freund's Adjuvant (IFA) (Sigma), respectively. 12-week-old mice were injected subcutaneously with 100 μg of CFA-OVA. After two weeks, mice received a subsequent immunization with 100 μg of IFA-OVA. For GC analysis, spleen was isolated on Day 7 post-immunization. GC staining was carried out with PNA-biotin (Vector, Burlingame, CA).

**Flow cytometry**

Single-cell suspensions of BM cells, splenocytes or PB cells were stained using standard procedures. Cells were adjusted to 2×10^7^ cells/ml in PBS with 2% FBS and 0.1% sodium azide, and 1×10^6^ cells were incubated 30 min on ice with appropriately diluted antibodies (1 ng/μl) in a total volume of 100 μl. After washing three times, the stained cells were analyzed using a flow cytometer (BD FACSAria). Processed samples were analyzed using CellQuest software (BD Biosciences). Anti-B220-APC/FITC, anti-Gr-1-APC, anti-CD3-PE/FITC, anti-Ter119-PE, anti-CD41-PE, anti-CD43-FITC, anti-IgM-FITC/APC, anti-IgD-PE, anti-CD21-PE and anti-CD23-FITC were purchased from eBioscience. Anti-CD16/CD32, anti-lambda 5-biotin and streptavidin-APC were purchased from BD Pharmingen.

**Plasmids and Constructs**

The *Gpr97* expression plasmid was based on the eukaryotic expression vector pcDNA^TM^3.1/*myc*-His (-) B (Invitrogen) by placing the cDNA insert under the control of the cytomegalovirus promoter. The *Gpr97* cDNA was created by PCR using the mouse BM cDNA as a template and cloned into the *Hind*III-*Bam*HI sites of pcDNA^TM^3.1/*myc*-His (-) B. The primers used were 5′- TATAAGCTTGCCACCATGCATCATCATCATCATCATTGAGCACTGATGGCGAC-3′ (*Gpr97* sense) and 5′ TTAGGATCCAATGGGAACTTTGAAGCC-3′ (*Gpr97* anti-sense). The *lambda 5* promoter was cloned by PCR using primers amplifying a 722-bp fragment containing sequences from nucleotide -613 to *+*109. The primers (sense: 5′-TAAGGTACCTATATGTCACAGGCTGGCCTTGA-3′; anti-sense: 5′-TATCTCGAGGACCCTCAAGTCCAAAG-3′) were included restriction sites to facilitate the cloning of the promoter into the *Kpn*I and *Xho*I sites of the luciferase reporter vector, pGL3 basic (Promega).

**Transient transfection and luciferase assay**

A total of 4×10^5^ Hela cells were grown overnight in 2 ml of complete DMEM medium in a 6-well plate. Empty vector pcDNA^TM^3.1/*myc*-His (-) B was used as a negative control, and plasmid pRL-TK (Promega), encoding *Renilla* luciferase, was contransfected as an internal control. 0.5 or 1 μg of *Gpr97* expression plasmid, 0.5 or 1 μg of pGL3-*lambda 5* vector and 0.1 μg of pRL-TK vector were cotransfected for each well using Lipofectamine 2000 (Invitrogen). Forty-eight hours after transfection, cells were lysed in a positive lysis buffer and assayed for luciferase activity using the Dual Luciferase Reporter System (Promega) with a Turner Design TD 20/20 Luminometer (Sunnyvale). Luciferase signal from the *lambda 5* promoter-luciferase construct was normalized to the *Renilla* luciferase signal to control the variation of transfection efficiency. Data presented were from two independent experiments.

**The antibodies used for Western blotting**

The primary antibodies are as follows: Gpr97 (1:300, Santa Cruz), Gapdh (1:10,000, KangChen), pCREB (1:1000, Cell Signaling), CREB (1:1000, Cell Signaling), p50 (1:1000, Cell Signaling), p65 (1:1000, Cell Signaling), β-actin (1:1000, Santa Cruz), or Lamin B (1:1000, Santa Cruz). IRDye 800CW-conjugated goat anti-rabbit IgG (1:10,000, LI-COR), rabbit anti-goat IgG (1:10,000, LI-COR) and goat anti-mouse IgG (1:10,000, LI-COR) were used as the secondary antibodies.

**List of primers used in this study**

| **Gene name** | **Direction** | **Sequence** |
| --- | --- | --- |
| *Gpr97* | Forward | 5′-caccttcgacttgaatgacactgctc-3′ |
|  | Reverse | 5′-tgctgatgttctggatcaatgcctt-3′ |
| *Ebf* | Forward | 5′- ATCCTGAAGAGAGCTGCCGACATT-3′ |
|  | Reverse | 5′- ACCTTGATTGGTGGCTTGTGATGC-3′ |
| *Sox4* | Forward | 5′- GGTTTCCAGTTCTTGCACGCTGTT-3′ |
|  | Reverse | 5′- TTGCAAGGTAGGAAGCCAAGAAGC-3′ |
| *E2A* | Forward | 5′- TTCAGCATGATGTTCCCGCTACCT-3′ |
|  | Reverse | 5′- TATGTCCGGCTAGGGTCAAAGGAA-3′ |
| *Ikaros* | Forward | 5′- TGAGGGTCAAGACATGTCCCAAGT-3′ |
|  | Reverse | 5′- TCACTCTTGGAGTTCTGCTGTGCT-3′ |
| *IL-7R* | Forward | 5′- ATTCACTGGCACATACAAGCGTGC-3′ |
|  | Reverse | 5′- TCTCCATCCTGGGCATTTCCACTT-3′ |
| *RAG-1* | Forward | 5′- TGGAGCAAGGTAGCTTAGCCAACA-3′ |
|  | Reverse | 5′- TTCTCCTTCTGTGCTTCTTCGGGT-3′ |
| *RAG-2* | Forward | 5′- TGACTCTTCCCCAAGTGCCGA-3′ |
|  | Reverse | 5′- CTTCCTGCTTGTGGATGTGAAATACTC-3′ |
| *Pax-5* | Forward | 5′- GTCAGCCATGGTTGTGTCAGCAAA-3′ |
|  | Reverse | 5′- GGAGTGGCAACCTTTGGTTTGGAT-3′ |
| *P13K* | Forward | 5′- AAGAACAATGCCAAACCCAGGAGC-3′ |
|  | Reverse | 5′- CCTGCTTCTTCAAGTCTTCTTCCAACC-3′ |
| *λ5* | Forward | 5′- TCACAATCCTAGGTCAGCCCAAGT-3′ |
|  | Reverse | 5′- TCTGTTTGGAGGGTTGGGTTGTCT-3′ |
| *VpreB1* | Forward | 5′- TCATGCTGCTGGCCTATCTCACA-3′ |
|  | Reverse | 5′- ATGGTGGCTCCAAGGGAAGAAGAT-3′ |
| *VpreB2* | Forward | 5′- TGGTCAGGTCCCAGGAGCAGTG-3′ |
|  | Reverse | 5′- ATGGTGGCTCCAAGGGAAGAAGAT-3′ |
| *IRF-4* | Forward | 5′- GAGCTGCAAGTGTTTGCTCACCAT-3′ |
|  | Reverse | 5′- ACAGTTGTCTGGCTAGCAGAGGT-3′ |
| *IRF-8* | Forward | 5′- TGATCGAACAGATCGACAGCAGCA-3′ |
|  | Reverse | 5′- TGGAAGCATCCACCTCCTGATTGT-3′ |
| *Lef-1* | Forward | 5′- AAGGCCAGAGAACACCCTGATGAA-3′ |
|  | Reverse | 5′- CTGCACCACGGGCACTTTATTTGA-3′ |
| *OBF-1* | Forward | 5′- TGTTCGAGTCAAGGAGCCAGTGAA-3′ |
|  | Reverse | 5′- AGCCAGCACATAATGTTCCCTCCT-3′ |
| *Oct-2* | Forward | 5′- CACCGAAAGAAATGGACCCGACA-3′ |
|  | Reverse | 5′- GCTGAATCGCCACTGGGGTCTT-3′ |
| *p105* | Forward | 5′- CATGGCAGACGATGATCCCTACG-3′ |
|  | Reverse | 5′- ATTTGAAGGTATGGGCCATCTGTTGA-3′ |
| *p100* | Forward | 5′- AGCAGTGTTCAGAGTTGGGAGTGT-3′ |
|  | Reverse | 5′- GGATCATAATCTCCATCATGTTCTTCTT-3′ |
| *c-Rel* | Forward | 5′- GCCGTGTGAACAAGAACTGTGGAA-3′ |
|  | Reverse | 5′- TTGTGAAAAAACACCTCTGGCTTCC-3′ |
| *RelA* | Forward | 5′- TGTGGAGATCATCGAACAGCCG-3′ |
|  | Reverse | 5′- TTCCTGGTCCTGTGTAGCCATTGAT-3′ |
| *RelB* | Forward | 5′- GCCGAATCAACAAGGAGAGCG-3′ |
|  | Reverse | 5′- CATCAGCTTGAGAGAAGTCGGCA-3′ |
| *Btk* | Forward | 5′- GGTGTCGTGAAATATGGGAAGTGGAG-3′ |
|  | Reverse | 5′- GCCATACAACTGCAVCAGCTTCTCAT-3′ |
| *Pten* | Forward | 5′- CATAACCCACCACAGCTAG-3′ |
|  | Reverse | 5′- GCAGACCACAAACTGAGG-3′ |
| *Blimp-1* | Forward | 5′-TTTGTGGACAGAGGCCGAGTTTG-3′ |
|  | Reverse | 5′- CAAAGCGTGTTCCCTTCGGTATGT-3′ |
| *Spi-B* | Forward | 5′- TGCAAGCCCTTCAGTTACCCAGATT-3′ |
|  | Reverse | 5′- AGGCTTCATAGGGAGCGATGGC-3′ |
| *Aiolos* | Forward | 5′-AAGCCGTACAAGTGTGAGTTCTGCG-3′ |
|  | Reverse | 5′-TCCCATCTCGGCTTTGATGTGTCT-3′ |
| *β–actin* | Forward | 5′- TACCCAGGCATTGCTGACAGG-3′ |
|  | Reverse | 5′- ACTTGCGGTGCACGATGGA -3′ |

1. **Supplementary Figures**

**
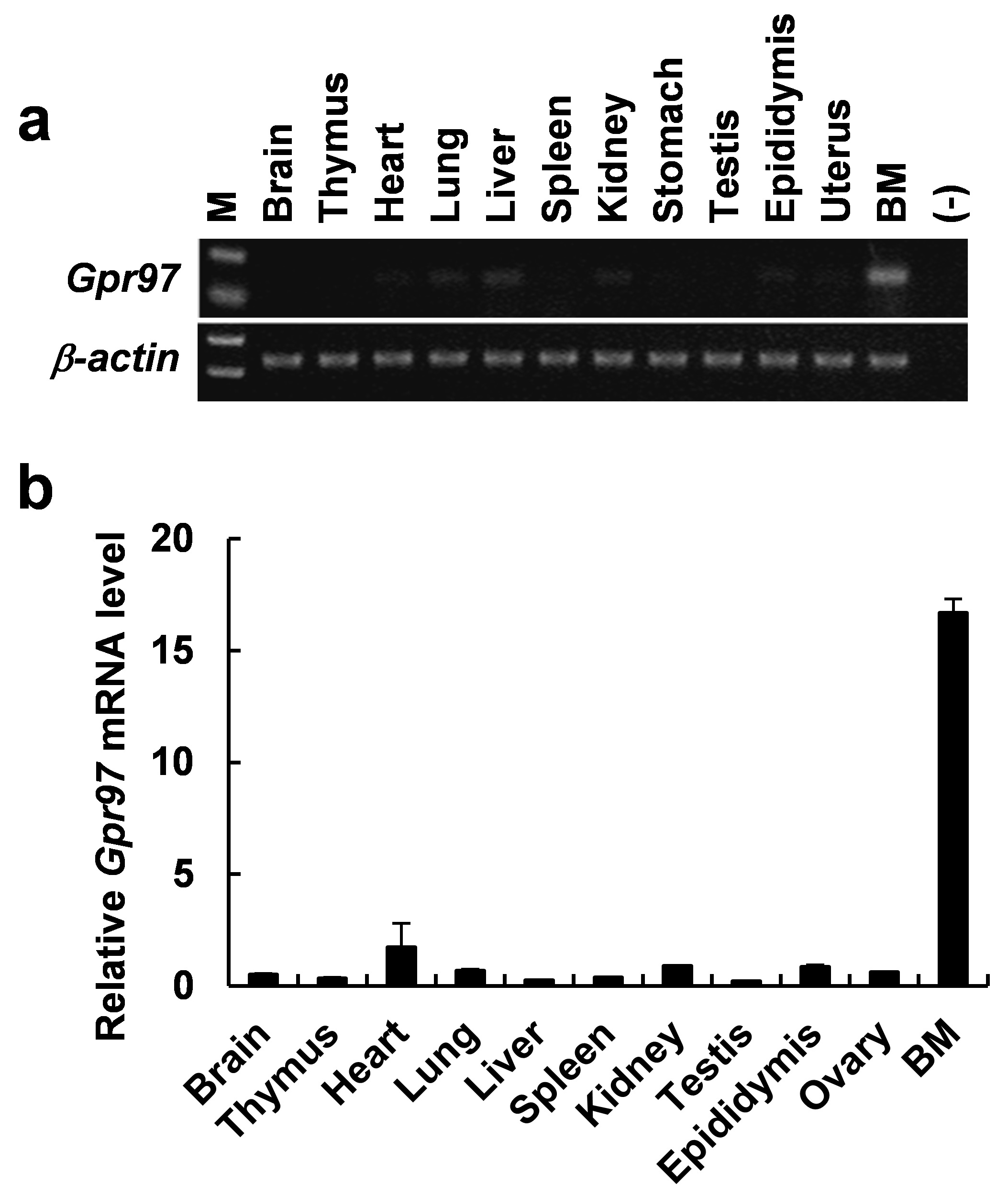
**

**Figure S1**. **Expression profile of *Gpr97* in normal mouse tissues.** Indicated tissues from adult WT mice were analyzed for *Gpr97* expression by semi-quantitative RT-PCR **(a)** and quantitative real-time PCR **(b)**. β-actin was shown as an internal control. Each bar represents the mean of triplicate measurements from a representative experiment, and error bars are standard errors. M: marker lane; (-): negative control without template.


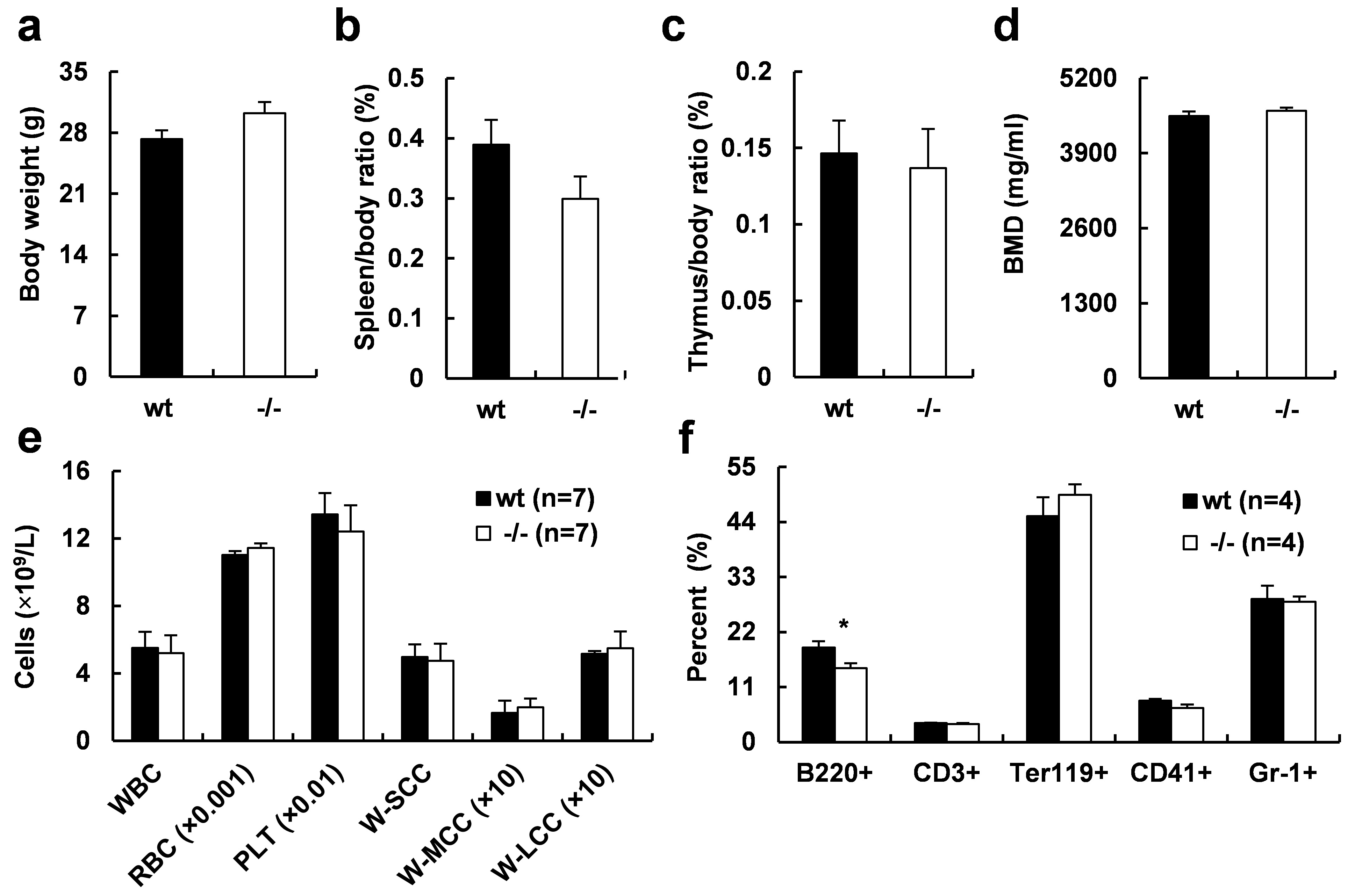


**Figure S2.** **B220^+^ cells were decreased in BM of Gpr97^-/-^ mice.** **(a)** Body weight of KO and control mice. **(b)** Spleen weight to body weight ratio of KO and control mice. **(c)** Thymus weight to body weight ratio of KO mice and control mice. **(d)** Bone mineral density of KO and control mice. Data show the mean and SEM. **(e)** Peripheral blood counts for WT and KO littermates (n=7 for each group). WBC indicates total white blood cell count; RBC, red blood cells (×10^-3^ for display purpose); PLT, platelets (×10^-2^); W-SCC, lymphocyte; W-MCC, intermediate cells (×10); W-LCC, granulocytes (×10). **(f)** BM cells were recovered from 12-wk-old WT (n=4) and age-, sex-matched KO mice (n=4). The cells were stained with mAbs as indicated and subjected to flow cytometric analysis. Data show the mean and SEM. The differences between WT and KO mice were examined for statistical significance that is indicated as asterisks: *, *p* < 0.05.


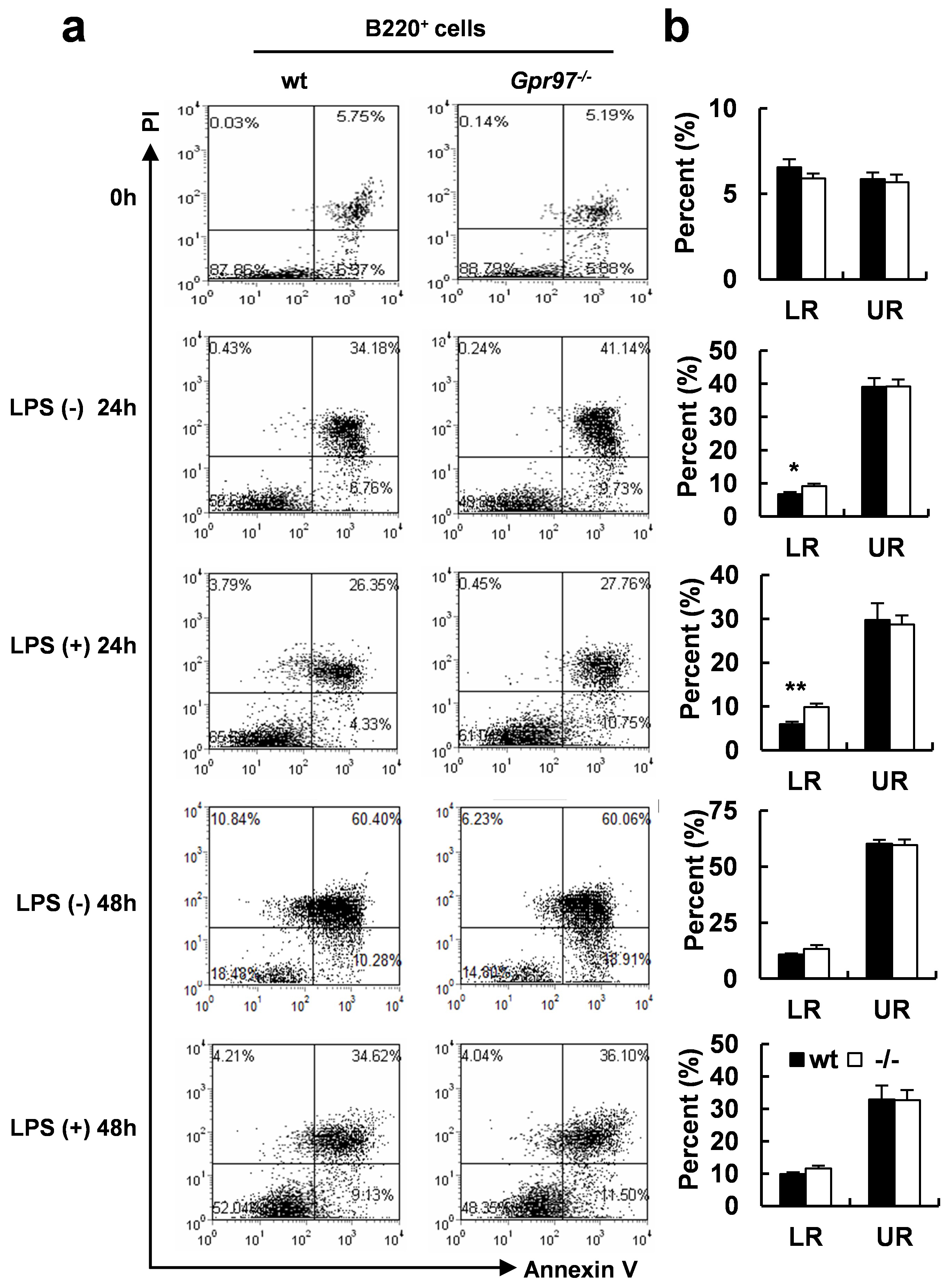


**Figure S3. Analysis of apoptosis in B220^+^ splenocytes of KO mice.** **(a)** Annexin V/PI staining and FACS analyses were performed to quantify phosphatidylserine externalization and advanced stages of cell death in uncultured, cultured splenocytes with or without LPS treatment for 24h or 48h. Cells in the lower right (LR) quadrant indicate annexin V^+^ PI^-^, early apoptotic cells. The cells in the upper right (UR) quadrant indicate annexin V^+^ PI^+^, late apoptotic cells, necrotic cells, or dead cells. **(b)** The bar charts summarize the relative numbers of annexin V^+^ PI^-^ cells (LR), annexin V^+^ PI^+^ cells (UR), and are expressed as mean (SEM, n=5). Individual fractions defined in (A) were used for calculations. The differences between WT and KO mice were examined for the statistical significance that is indicated as asterisks: *, *p* < 0.05; **, *p* < 0.01.

**
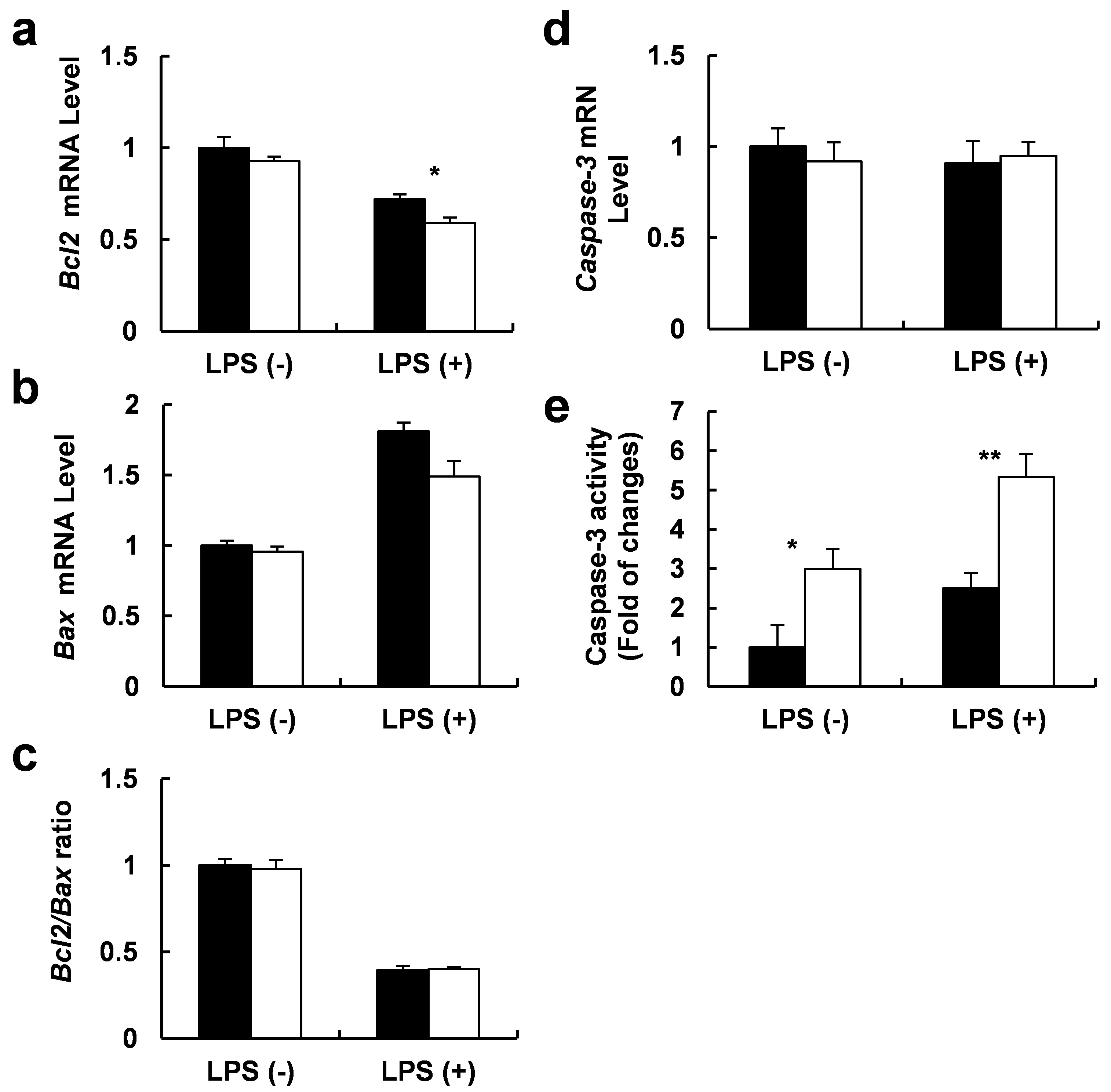
**

**Figure S4. Caspase-3 activity was increased in splenic B220^+^ cells lacking Gpr97.** Total RNA was extracted from splenic B220^+^ cells of WT and KO mice (n=3 for each) cultured with or without LPS for 24h. The results of real time qPCR for Bcl-2 **(a)** and Bax **(b)** mRNA levels are shown. **(c)** The ratio of Bcl-2/Bax mRNA is shown. **(d)** Caspase-3 mRNA level was analyzed by real time qPCR as above. **(e)** Caspase-3 activity is increased in B220^+^ lymphocytes cultured with or without LPS for 24h. Data show the mean and SEM. The differences between WT and KO mice were examined for statistical significance that is indicated as asterisks: *, *p* < 0.05; **, *p* < 0.01.
